# Extending Helminth Control beyond STH and Schistosomiasis: The Case of Human Hymenolepiasis

**DOI:** 10.1371/journal.pntd.0002321

**Published:** 2013-10-24

**Authors:** Ricardo J. Soares Magalhães, Cláudia Fançony, Dina Gamboa, António J. Langa, José Carlos Sousa-Figueiredo, Archie C. A. Clements, Susana Vaz Nery

**Affiliations:** 1 University of Queensland, Infectious Disease Epidemiology Unit, School of Population Health, Herston, Queensland, Australia; 2 Centro de Investigação em Saúde em Angola, Caxito, Rua Direita do Caxito, Hospital Provincial do Bengo, Caxito, Angola; 3 Disease Control Strategy Group, Liverpool School of Tropical Medicine, Liverpool, United Kingdom; 4 Department of Infectious and Tropical Diseases, London School of Hygiene & Tropical Medicine, London, United Kingdom; London School of Hygiene & Tropical Medicine, United Kingdom

The WHO recently produced updated guidelines for managers of helminth control programmes, specifically targeting soil-transmitted helminthiasis (STH) and schistosomiasis in school-age children [1]. In the case of schistosomiasis, this strategic document advocates treatment with praziquantel (PZQ) as the cornerstone of control, with the objective of reducing infection-associated morbidity, which is estimated to be 70 million disability-adjusted life years (DALYs). There are, however, other helminth infections currently absent from these guidelines that can result in important morbidity effects in children and are also treatable with PZQ. An important example is hymenolepiasis, which is caused by a cyclophyllidean tapeworm from the genus *Hymenolepis*.

Hymenolepiasis was first recognised in the small intestine of a boy in Cairo in 1851 by Bilharz [2]. The two species of *Hymenolepis* infecting man, namely *H. nana* and *H. diminuta*, are ubiquitous and *H. nana* is by far the most common of the two parasites. *H. nana* infections are considered to be the most prevalent human cestodiasis in the world [2]–[4]. Studies over the past 50 years documenting the prevalence of *H. nana* indicate that in some communities this infection can reach prevalence as high as 21% in children (see Text S1). Cases of hymenolepiasis are often seen as clusters within a family and in institutions where children are crowded together (e.g., orphanages, childcare centres, and boarding schools) [2], [5]–[7], suggesting a common source of exposure. The majority of infections occur as autoinfections as a result of contamination of food or water by humans, usually children, excreting viable eggs in their faeces [4]. While most *H. nana* infections are usually asymptomatic, numerous studies have documented that heavy infections with *H. nana* can cause severe morbidity in children, including severe diarrhoea, abdominal pain, decreased appetite, irritable behaviour, anal or nasal pruritus, and reduced growth [2], [4], [7]–[9].

Similarly to schistosomiasis, a single dose of PZQ eliminates the vast majority of *Hymenolepis* egg excretion [10]–[12]. However, it is likely that a PZQ gap exists in many communities being targeted by MDA in that the geographical location of *Hymenolepis* infections may not necessarily overlap with that of schistosomiasis. Guiding PZQ delivery solely on the basis of the distribution of schistosomiasis may miss communities endemic to *Hymenolepis* infection also in need of PZQ. This means that the populations at risk of hymenolepiasis may need to also be identified so that PZQ delivery can be extended to those areas.

In order to test our proposition, we have analysed data from a parasitic disease survey of 2,168 children aged ≤15 years, including 1,098 girls and 1,070 boys in the Dande municipality in Northern Angola. Previous analysis of this dataset revealed that children were at significantly increased risk of *H. nana* infection (prevalence of 6.2% [95% CI: 4.9–7.8%] in preschool children and 7.3% [95% CI: 5.8–9.0%] in school-age children) compared to adults (prevalence of 1.9% [95% CI: 1.1–3.1%]) [13]. Using these data, we aimed to describe the epidemiology of *H. nana* infection by quantifying the role of individual and household factors and the physical environment (land surface temperature, distance to irrigation canals and rivers) in *H. nana* infection; quantify the role of *H. nana* infection on morbidity outcomes such as anaemia, diarrhoea, abdominal pain, and growth; quantify the geographical variation in *H. nana* infection prevalence in children aged ≤15 years; generate the first high-resolution *H. nana* infection map; and compare this map with a preexisting *S. haematobium* map for the region [14] to identify the limitations of targeting PZQ distribution on the basis of urogenital schistosomiasis alone.

*H. nana* transmission is known to be facilitated by contact with environments contaminated with human faeces, use of inadequate drinking sources, the absence of proper sanitation and ineffective treatment of excreta or waste, deficient personal hygiene, and the presence of another infected person in the household [5], [7], [15]–[17]. In line with previous studies, we found that bathing in irrigation canals is an important risk factor for *H. nana* infection. The irrigation canals are a legacy of the sugar plantation industry set up in the 1950s and surround the provincial capital of Caxito and neighbouring communities. While the sugar mill is no longer in production, the irrigation canals are used by the population for their daily necessities including clothes washing, recreation, and in some instances as a source of drinking water [18]. It has been shown that overcrowded conditions contribute to an increased risk of *H. nana* infection in children due to a deterioration of the general hygiene situation of the household, which increases faecal-oral transmission of *H. nana* [4]. Our results indicate that overcrowding is likely to be an important contributor to *H. nana* infection in that the risk is increased in households with more rooms probably due to the resulting lower hygiene score. This finding is also consistent with the view that hymenolepiasis is more often seen as clusters within a family [2].The results from our study also demonstrate a possible foodborne source for *H. nana* infection in that households that reported not washing their vegetables were at increased risk of infection compared to those that do wash their vegetables. This finding is corroborated by a recent study reporting isolation of *H. nana* eggs from raw vegetables [19].

While *H. nana* prevalence in children aged <5 years was lower compared to children aged ≥5–15 years, our results suggest an association between *H. nana* infection and previous history of abdominal pain, and *H. nana* and *T. trichiura* coinfections to acute malnutrition in children aged <5 years. We did not see an independent effect of *T. trichiura* infection on morbidity. The effect on morbidity identified in this study is consistent with the known pathophysiology of *H. nana* and *T. trichiura* worms, which are known to cause inflammation, bleeding, and dysentery through mucosal injury and local, humoral, and cellular responses to infection [4], [20], [21]. The exacerbated morbidity profiles observed in children aged <5 years compared to older children may be a result of the absence of acquired immunity to helminth infections. In addition, the fact that children <5 years are at increased risk of morbidity is of concern because dose poles are not available for this age group and PZQ delivery for schistosomiasis is aimed at school-age children rather than pre-school children. The fact that *H. nana* and *T. trichiura* coinfections are also associated with previous history of abdominal pain and acute malnutrition is a reasonable argument to advocate the delivery of PZQ to the communities with the aim of reducing helminth-associated morbidity in the study area. While albendazole may be made available to this population due to the high endemicity of STHs (<30%), the high spatial heterogeneity of *S. haematobium* endemicity in the area means that PZQ will not be made available to all communities on an annual basis [13]. The results of our study show that guiding delivery of PZQ solely on schistosomiasis in integrated programmes that also include albendazole is likely to overlook the important interaction of STH with other parasites such as *H. nana*, which should be the focus of interventions even in areas of low endemicity.

The prevalence of hymenolepiasis in a community can be a useful indicator of the degree of faecal contamination of an environment and/or the level of hygiene practice. Because WASH coverage in sub-Saharan Africa shows considerable regional disparities [22]–[24], the disease burden due to *H. nana* infection is likely to be highly geographically variable. Modern geographical risk prediction methods using model-based geostatistics (MBG) provide an extensive set of spatial modeling tools for assessing the geographical overlap of multiple parasite infections and are being used as control tools for targeting helminth interventions [25]. One approach is overlaying prevalence of infection maps for multiple parasites (i.e., coendemicity mapping). To that regard, our predictive map of *H. nana* infection showed an area of high *H. nana* risk (prevalence >8%) associated with more populated areas near and around Caxito and a large cluster predicted to the commune of Mabubas that is unrelated to the endemicity of schistosomiasis (Figure 1). The fact that areas likely to receive PZQ annually or biannually (due to high to moderate *S. haematobium* infection, respectively) do not completely overlap with areas of high *H. nana* prevalence of infection may pose an import gap in PZQ delivery needs (Figure 1). Furthermore, PZQ may not be sufficiently efficacious to eliminate *H. nana* infection, as effective treatment sometimes requires prolonged therapy with niclosamide (5–7 days) to eliminate emerging adult worms and to eradicate the infection [3].

**Figure 1:**
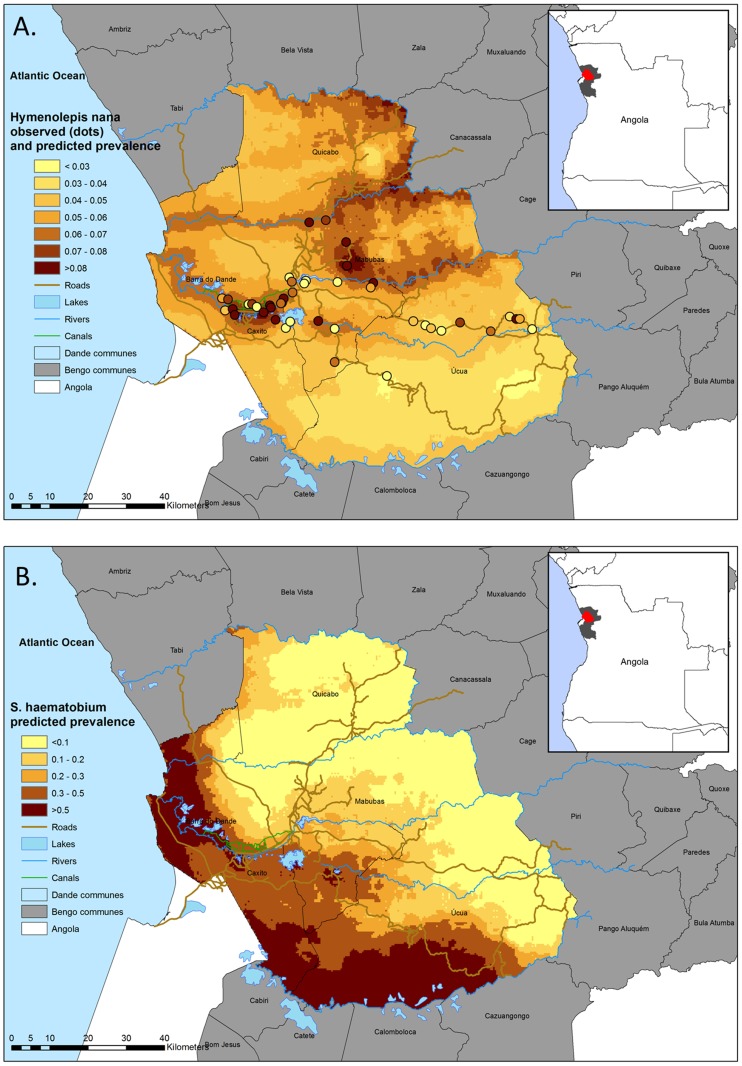
Observed and predicted prevalence of *Hymenolepis nana* infection (A) and predicted prevalence of *S. haematobium* (B) in the Dande municipality in Angola.

The results highlight the need for WASH improvements to be delivered to communities concomitantly with anthelminth therapy if resources are available. The impact of autoinfection is unlikely to change unless WASH interventions are put in place. More importantly, in this study we show for the first time that *H. nana* infection is an important contributor to infection-associated morbidity, particularly in children aged <5 years, and that the delivery of PZQ to control schistosomiasis and hymenolepiasis should take into consideration their coendemicity. If delivery of PZQ is based solely on schistosomiasis endemicity thresholds, areas in need of PZQ to treat *H. nana* infections will be reached at very low frequencies or not at all. However, it remains to be demonstrated whether targeting of communities for PZQ distribution on the basis of *H. nana* disease burden is likely to be cost-effective, and further economic analysis needs to be conducted. To improve visibility and enhance advocacy for the control of hymenolepiasis, it may be warranted to include this infection in the list of neglected tropical diseases.

## Supporting Information

Text S1 Technical information.
(DOC)
