# Supplementary material for: Extending Helminth Control beyond STH and Schistosomiasis: The Case of Human Hymenolepiasis
Source: PLoS Negl Trop Dis. 2013 Oct 24;7(10):e2321. doi: 10.1371/journal.pntd.0002321 (PMC3812097; doi:10.1371/journal.pntd.0002321)
Supplement: Text S1 — Technical information. (DOC) [file pntd.0002321.s001.doc]

**Extending helminth control beyond STH and schistosomiasis: the case of human hymenolepiasis**

**Text S1**

*Table S1Review of studies reporting Hymenolepis* nana infection

| **Study** | **Year of survey** | **Location** | **Population** | **Prevalence** |
| --- | --- | --- | --- | --- |
| [Asian Pac J Trop Med.](http://www.ncbi.nlm.nih.gov/pubmed/21771685) 2011 May;4(5):401-3. Epub 2011 Jun 22. | 2009 | Burkina Faso | Patients | 2.25% |
| [East Mediterr Health J.](http://www.ncbi.nlm.nih.gov/pubmed/20214122) 2009 Sep-Oct;15(5):1098-104. | 2007 | Sudan | Food-handlers | 1.6% |
| [Ethiop Med J.](http://www.ncbi.nlm.nih.gov/pubmed/19743775) 2009 Jan;47(1):9-16. | 2007 | Ethiopia | Schoolchildren | 4.7% |
| [J Helminthol.](http://www.ncbi.nlm.nih.gov/pubmed/19712536) 2010 Jun;84(2):132-5. Epub 2009 Aug 28. | 2008 | Sudan | Schoolchildren | 5.2% |
| [J Egypt Soc Parasitol.](http://www.ncbi.nlm.nih.gov/pubmed/19621655) 2009 Apr;39(1 Suppl):371-81. | 2007 | Egypt | Community rural | 2.96% |
| [Ann Biol Clin (Paris).](http://www.ncbi.nlm.nih.gov/pubmed/19297294) 2009 Mar-Apr;67(2):191-202. | 1996-2005 | Morocco | Community | 2.68% |
| [J Egypt Soc Parasitol.](http://www.ncbi.nlm.nih.gov/pubmed/19143135) 2008 Apr;38(1):255-64. | 2006 | Egypt | Community | 0.38% |
| [J Egypt Soc Parasitol.](http://www.ncbi.nlm.nih.gov/pubmed/18383779) 2007 Dec;37(3):775-84. | 2005 | Lybia | Schoolchildren | 6% |
| [J Egypt Soc Parasitol.](http://www.ncbi.nlm.nih.gov/pubmed/17580578) 2007 Apr;37(1):205-14. | 2005 | Lybia | Schoolchildren | 0.1% |
| [J Egypt Soc Parasitol.](http://www.ncbi.nlm.nih.gov/pubmed/16927862) 2006 Aug;36(2):467-80. | 2004 | Egypt | Community | 2.2% |
| [Ann Trop Med Parasitol.](http://www.ncbi.nlm.nih.gov/pubmed/16762112) 2006 Jun;100(4):315-26. | 2003-2004 | Uganda | Preschool | 11% |
| [Afr Health Sci.](http://www.ncbi.nlm.nih.gov/pubmed/16246001) 2005 Sep;5(3):276-80. | 2004 | South Africa | Schoolchildren | 2.2% |
| [Ethiop Med J.](http://www.ncbi.nlm.nih.gov/pubmed/15296415) 2003 Oct;41(4):333-44. | 1999-2002 | Ethiopia | Community | < 4% |
| [Bull Soc Pathol Exot.](http://www.ncbi.nlm.nih.gov/pubmed/14582293) 2003 Aug;96(3):187-90. | 1997-1999 | Mali | Schoolchildren | < 1% |
| [Ann Trop Med Parasitol.](http://www.ncbi.nlm.nih.gov/pubmed/12662420) 2003 Jan;97(1):31-5. | 1998 | Mozambique | Children | 1.1% |
| [Mem Inst Oswaldo Cruz.](http://www.ncbi.nlm.nih.gov/pubmed/11784922) 2001 Nov;96(8):1055-9. | 1997-1998 | Nigeria | Pregnant Nigerian women | 1.6% |
| [Tunis Med.](http://www.ncbi.nlm.nih.gov/pubmed/10894046) 2000 Feb;78(2):109-14. | 1997 | Morocco | Children | 7.2% |
| [Bull Soc Pathol Exot.](http://www.ncbi.nlm.nih.gov/pubmed/9264753) 1997;90(1):51-4. | 1994 | Cote D’Ivoire | Schoolchildren | 1.1% |
| [Bol Chil Parasitol.](http://www.ncbi.nlm.nih.gov/pubmed/9830722) 1998 Jan-Jun;53(1-2):31-4. | 1981-1995 | Cuba | Community | 0.008% |
| [Am J Clin Pathol.](http://www.ncbi.nlm.nih.gov/pubmed?term=Am J Clin Pathol 1995%2C 104(3)%3A272-278) 1995 Sep;104(3):272-8. | 1989-1992 | Canada | children | 2% |
| [Rev Inst Med Trop Sao Paulo.](http://www.ncbi.nlm.nih.gov/pubmed?term=Rev Inst Med Trop Sao Paulo 1995%2C 37(6)%3A501-506) 1995 Nov-Dec;37(6):501-6. | 1993 | Brazil | Children in day-care centres | 8.8% |
| [West Afr J Med.](http://www.ncbi.nlm.nih.gov/pubmed/7626531) 1995 Jan-Mar;14(1):39-42. | 1993 | Nigeria | Community | 0.4% |
| [Korean J Parasitol.](http://www.ncbi.nlm.nih.gov/pubmed?term=Korean J Parasitol 1994%2C 32(1)%3A27-33) 1994 Mar;32(1):27-33. | 1984-1992 | Korea | Patients | 0.03% |
| [J Egypt Soc Parasitol.](http://www.ncbi.nlm.nih.gov/pubmed/8169435) 1994 Apr;24(1):137-45. | 1992 | Egypt | Children - orphanages | 2% |
| [J Parasitol.](http://www.ncbi.nlm.nih.gov/pubmed?term=J Parasitol. 1994 Apr%3B80(2)%3A245-50.) 1994 Apr;80(2):245-50. | 1992 | Zimbabwe | Schoolchildren | 21% (24% in rural and 18% in urban areas) |
| [Rev Gastroenterol Peru.](http://www.ncbi.nlm.nih.gov/pubmed?term=Rev Gastroenterol Peru 1991%2C 11(3)%3A153-160.) 1991;11(3):153-60. | 1990 | Peru | Preschool children | 21% |
| [Ann Trop Med Parasitol.](http://www.ncbi.nlm.nih.gov/pubmed/1463359) 1992 Aug;86(4):387-93. | 1990 | Sudan | Refugees based in Juba | 11% |
| [East Afr Med J.](http://www.ncbi.nlm.nih.gov/pubmed/1396210) 1992 Aug;69(8):437-41. | 1988 | Kenya | Preschool - diarrheal | 3.8% |
| [Ethiop Med J.](http://www.ncbi.nlm.nih.gov/pubmed/1396614) 1992 Jul;30(3):129-34. | 1987 | Ethiopia | Community | 0.6% |
| [MMWR CDC Surveill Summ.](http://www.ncbi.nlm.nih.gov/pubmed?term=MMWR CDC Surveill Summ 1991%2C 40(4)%3A25-45) 1991 Dec;40(4):25-45. | 1987 | United States | Community | 0.4% |
| [Trans R Soc Trop Med Hyg.](http://www.ncbi.nlm.nih.gov/pubmed?term=Transactions of the Royal Society of Tropical Medicine and Hygiene 1991%2C 85(1)%3A70-73.) 1991 Jan-Feb;85(1):70-3. | 1989 | Honduras | Rural children | 8% |
| [J Egypt Soc Parasitol.](http://www.ncbi.nlm.nih.gov/pubmed/2033304) 1991 Apr;21(1):293-300. | 1988 | Egypt | Children | 16% |
| [Acta Trop.](http://www.ncbi.nlm.nih.gov/pubmed/1671621) 1991 Jan;48(3):195-202. | 1989 | Saudi Arabia | Schoolchildren | 3.0% |
| [Ann Pediatr (Paris).](http://www.ncbi.nlm.nih.gov/pubmed/2624382) 1989 Dec;36(10):669-701. | 1986 | Niger | Children | 10.8% |
| [Ethiop Med J.](http://www.ncbi.nlm.nih.gov/pubmed/2513182) 1989 Oct;27(4):183-91. | 1986 | Ethiopia | Community | 0.6% |
| [Trans R Soc Trop Med Hyg.](http://www.ncbi.nlm.nih.gov/pubmed/2617631) 1989 Sep-Oct;83(5):681-3. | 1985-1987 | Namibia | Patients | 1% |
| [Ethiop Med J.](http://www.ncbi.nlm.nih.gov/pubmed/2496975) 1989 Apr;27(2):73-83. | 1987 | Ethiopia | Community | < 3% |
| [Parassitologia.](http://www.ncbi.nlm.nih.gov/pubmed?term=Parassitologia 1988%2C 30(2-3)%3A263-269.) 1988 May-Dec;30(2-3):263-9. | 1986 | Bolivia | Community | 8.7% |
| [Parassitologia.](http://www.ncbi.nlm.nih.gov/pubmed/3508506) 1987 Apr;29(1):15-25. | 1983 | Democratic Republic of São Tomé and Principe | Community | 0.2% |
| [J Hyg Epidemiol Microbiol Immunol.](http://www.ncbi.nlm.nih.gov/pubmed/3701056) 1986;30(1):99-102. | 1984 | Niger Delta | Preschool | 18.3% |

*Table S2. Hymenolepiasis* in 2,062 children aged ≤15 years in Dande municipality, Angola

| **Variable** |  | **Hymenolepiasis** | |
| --- | --- | --- | --- |
|  | **Total** | **Yes** | **No** |
| **Total number** | 2,062 | 146 | 1,916 |
| **Gender** |  |  |  |
| Male | 1,007 | 69 | 938 |
| Female | 1,055 | 77 | 978 |
| **Infection intensity (eggs per gram of stool)** |  |  |  |
| Median |  | 264 | - |
| Range (minimum – maximum) |  | 12 – 23,724 | - |
| **Age in years** |  |  |  |
| Median |  | 6 | 6 |
| Range (minimum – maximum) |  | 1.5 – 16 | 0.5 – 16 |
| **Height in cm** |  |  |  |
| Median |  | 111 | 110 |
| Range (minimum – maximum) |  | 77 – 157 | 13.5 – 170 |
| **Weight in kg** |  |  |  |
| Median |  | 17.8 | 17 |
| Range (minimum – maximum) |  | 8 – 64 | 4 – 80 |
| **Weight-for-height z-score <-2** |  |  |  |
| No | 790 | 39 | 751 |
| Yes | 84 | 8 | 76 |
| **Height-for-age z-score <-2** |  |  |  |
| No | 1,389 | 106 | 1,283 |
| Yes | 661 | 40 | 621 |
| **Weight-for-age z-score <-2** |  |  |  |
| No | 1,250 | 86 | 1,164 |
| Yes | 377 | 28 | 349 |
| **Anaemia (Hb<110 g/L)** |  |  |  |
| No | 1,168 | 81 | 937 |
| Yes | 840 | 63 | 927 |
| **Diarrhoea** |  |  |  |
| No | 950 | 70 | 880 |
| Yes | 1,069 | 74 | 995 |
| **Previous history of abdominal pain** |  |  |  |
| No | 286 | 17 | 269 |
| Yes | 1,740 | 125 | 1,615 |
| **Ascariasis** |  |  |  |
| No | 1,731 | 117 | 1,614 |
| Yes | 331 | 29 | 302 |
| **Trichuriasis** |  |  |  |
| No | 1,839 | 124 | 1,715 |
| Yes | 223 | 22 | 201 |

*Table S3. Univariable and multivariable associations with Hymenolepis na*na infection in children aged ≤15 years in Dande municipality, Angola.

| **Variable** | **Univariable** | | | **Multivariable** | | |
| --- | --- | --- | --- | --- | --- | --- |
|  | **Coefficient**  **(95% CI)** | **p-value** | **overall**  **p-value** | **Coefficient**  **(95% CI)** | **p-value** | **Overall**  **p-value** |
| Age in years | 0.04 (0.004,0.08) | 0.031 |  | 0.04 (0.00,0.07) | 0.050 |  |
| Female (vs Male) | 0.079 (-0.23,0.37) | 0.655 |  | 0.07 (-0.24,0.38) | 0.642 |  |
| Received praziquantel  (vs didn't receive praziquantel) | -0.002 (-0.85,0.84) | 0.997 |  | - |  |  |
| Bathe in dam (vs doesn't bathe in dam) | -10.38 (-12.39,-8.36) | <0.001 |  | - |  |  |
| Bathe in lagoon (vs doesn't bathe in lagoon) | 0.02 (-0.56,0.60) | 0.948 |  | - |  |  |
| Bathe in river (vs doesn't bathe in river) | 0.22 (-0.22,0.66) | 0.325 |  | - |  |  |
| Bathe in irrigation canal (vs doesn't bathe in canal) | 0.43 (0.07,0.80) | 0.020 |  | 0.39 (0.02,0.76) | 0.038 |  |
| Bathe at home (vs doesn't bathe at home) | 0.10 (-0.41,0.61) | 0.704 |  | - |  |  |
| Wash hands (vs doesn't wash hands) | -0.25 (-0.61,0.11) | 0.175 |  | - |  |  |
| Use soap (vs doesn't use soap) | -0.39 (-0.76,-0.02) | 0.038 |  | - |  |  |
| Uses shoes (vs doesn't use shoes) | -0.31 (-0.66,0.04) | 0.082 |  | - |  |  |
| Wash vegetables (vs doesn't wash vegetables) | -0.67 (-1.03,-0.31) | <0.001 |  | -0.56 (-0.98,-0.13) | 0.010 |  |
| Owns animals (vs doesn't own animals) | 0.17 (-0.23,0.58) | 0.397 |  | - |  |  |
| Number of rooms in household | 0.14 (0.01,0.26) | 0.029 |  | 0.13 (0.01,0.25) | 0.037 |  |
| Well water (vs river, lagoon, canal) | -0.23 (-0.74,0.28) | 0.378 | 0.252 | -0.11 (-0.65,0.42) | 0.680 | 0.3295 |
| Pipped water (vs river, lagoon canal) | -0.27(-0.69,0.15) | 0.202 |  | -0.31 (-0.73,0.10) | 0.136 |  |
| Latrine with water (vs no latrine) | 0.10 (-0.46,0.65) | 0.731 | 0.879 | - |  |  |
| Latrine without water (vs no latrine) | 0.10 (-0.32,0.52) | 0.654 |  | - |  |  |
| Intercept | - |  |  | -2.75 (-3.39,-2.10) | <0.001 |  |

*Figure S1 Residual semivariogram of Hymenolepis nana infection.*


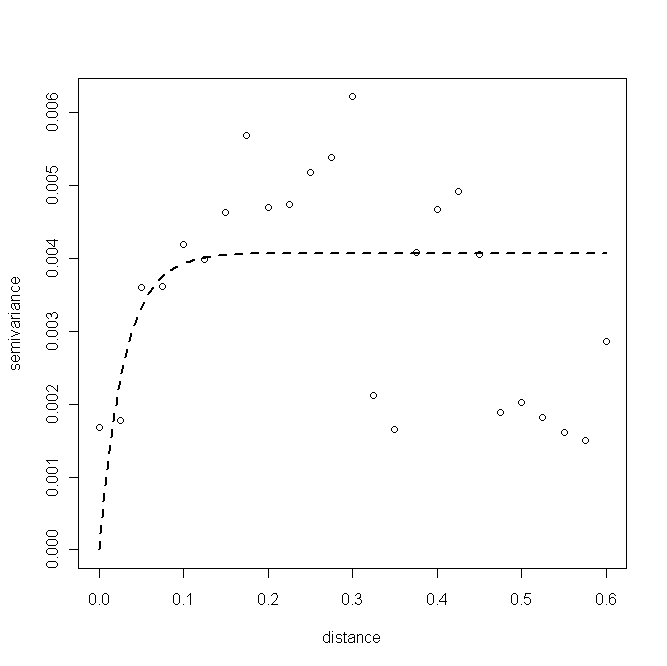


*Box S1. Model specifications*

For the purpose of Bayesian geostatistical modelling the *H. nana* infection status is considered a binary outcome variable *Y_i_* which was labelled *Y_i_* =1 for infected child and 0 for non-infected child. The models used assume a conditional Bernoulli model for the binary outcome variable where the probability of a child *i* being anaemic, given the location *j* of the child is given by:

*i*

*p*

*k*

*j*

*i*

*k*

*j*

*i*

*u*

*x*

*p*

*log*

+

×

+

=

∑

=

1

,

,

)

(

*β*

*α*

where *Y_i,j_* is the infection status of an child in location *j*, *p_i,j_* is the probability of an child being infected in location j, *α* is the intercept, *x_i,j_* is a matrix of covariates, *β* is a matrix of coefficients and *u_i_* is a geostatistical random effect defined by an isotropic powered exponential spatial correlation function:

,

where *d_ab_* are the distances between pairs of points *a* and *b*, and is the rate of decline of spatial correlation per unit of distance. Non-informative priors were used for *α* (uniform prior with bounds - and ) and the coefficients (normal prior with mean = 0 and precision = 1 × 10^-4^). The precision of *u_i_* was given a non-informative gamma distribution.

In all models, a burn-in of 5,000 iterations was allowed, followed by 10,000 iterations where values for the intercept, coefficients and predicted probability of infection at the prediction locations were stored. Diagnostic tests for convergence of the stored variables were undertaken, including visual examination of history and density plots; convergence was successfully achieved after 5,000 iterations.

The predictions of the prevalence of *H. nana* were made at the nodes of a 0.1 X 0.1 decimal degree grid (approximately 11 km^2^) by interpolating the geostatistical random effect and adding it to the sum of the products of the coefficients for the fixed effects and the values of the fixed effects at each prediction location. Values of predicted prevalence of *H. nana* infection at unsampled locations were stored for older female children. The interpolation of the random effect was done using the *spatial.unipred* kriging function in WinBUGS; the *spatial.unipred* command implements Bayesian kriging where the values of predicted prevalence at unsampled locations are estimated (interpolated) independently of neighbouring values, as opposed to joint prediction which is conditional on the values of neighbouring unsampled locations. Joint prediction was not considered feasible in this study due to being extremely intensive computationally.

*Table S4. Spatial effects for Hymenolepis nana prevalence in* children aged ≤15 years in Dande municipality, Angola

| **Variable** | **Posterior mean (95%Credible Interval)** |
| --- | --- |
| Age in years | 0.04 (-0.004,0.08) |
| Female (vs male) | 0.05 (-0.29,0.39) |
| Land surface temperature* | 0.12 (-0.31,0.66) |
| Distance to river* | -0.08 (-0.43,0.31) |
| Distance to irrigation canals* | -0.29 (-1.0,-0.02) |
| Intercept | -2.89 (-3.56,-2.13) |
| Rate of decay of spatial autocorrelation (φ) | 12.37 (3.32,19.62) |
| Variance of spatial random effect | 0.40 (0.003,1.62) |

*Variables were standardised to have mean = 0 and standard deviation = 1

*Table S5 Multivariable models of morbidity associated with Hymenolepis nana infection in children aged ≤5 years in Dande municipality, Angola*

| **Morbidity model** | **Coefficient (95% CI)** |
| --- | --- |
| **Anaemia (Hb<11.0 g/L)** |  |
| *Hymenolepis nana* | -0.25 (-0.92,0.43) |
| *Trichuris trichiura* | -0.64 (-1.20,-0.07)* |
| *Hymenolepis nana × Trichuris trichiura* | 1.13 (-1.27,3.52) |
| *Intercept* | 0.45 (0.25,0.65)* |
| **Diarrhoea** |  |
| *Hymenolepis nana* | 0.18 (-0.44,0.80) |
| *Trichuris trichiura* | -0.17 (-0.79,0.44) |
| *Hymenolepis nana × Trichuris trichiura* | 0.25 (-2.67,3.16) |
| *Intercept* | 0.44 (0.13,0.75) |
| **Previous history of abdominal pain** |  |
| *Hymenolepis nana* | 0.66 (-0.28,1.60) |
| *Trichuris trichiura* | 0.40 (-0.49,1.29) |
| *Hymenolepis nana × Trichuris trichiura* | 1.42 (0.10,1.29)* |
| *Intercept* | 1.48 (1.19,1.78)* |
| **Weight-for-height z-score <-2** |  |
| *Hymenolepis nana* | 0.85 (0.06,1.64)* |
| *Intercept* | -2.35 (-2.72,-1.99)* |
| **Height-for-age z-score <-2** |  |
| *Hymenolepis nana* | -1.03 (-1.70,-0.36)* |
| *Trichuris trichiura* | -0.51 (-1.16,0.14) |
| *Hymenolepis nana × Trichuris trichiura* | -1.23 (-1.39,-1.08)* |
| Intercept | -0.10 (-0.27,0.07) |
